# Dynamic Measurement of Protein Translation in Mycobacteria Using Nontargeted Stable Isotope Labeling in Combination with MALDI-TOF Mass Spectrometry-Based Readout

**DOI:** 10.1021/acs.analchem.4c03931

**Published:** 2025-02-25

**Authors:** Christoph Haisch, Anna-Cathrine Neumann-Cip, Axel Imhof, Andreas Schmidt, Ignasi Forne, Michael Hoelscher, Andreas Wieser

**Affiliations:** † Chair of Analytical Chemistry, Technical University of Munich, 85748 Garching, Germany; ‡ Division of Infectious Diseases and Tropical Medicine, LMU University Hospital, LMU Munich, 80802 Munich, Germany; § German Center for Infection Research (DZIF), Partner Site Munich, 80802 Munich, Germany; ∥ Chair of Medical Microbiology and Hospital Epidemiology, Max von Pettenkofer Institute, Faculty of Medicine, LMU Munich, 80802 Munich, Germany; ⊥ Biomedical Center, Protein Analysis Unit, Faculty of Medicine, Ludwig-Maximilians University Munich, 82152 Planegg-Martinsried, Germany

## Abstract

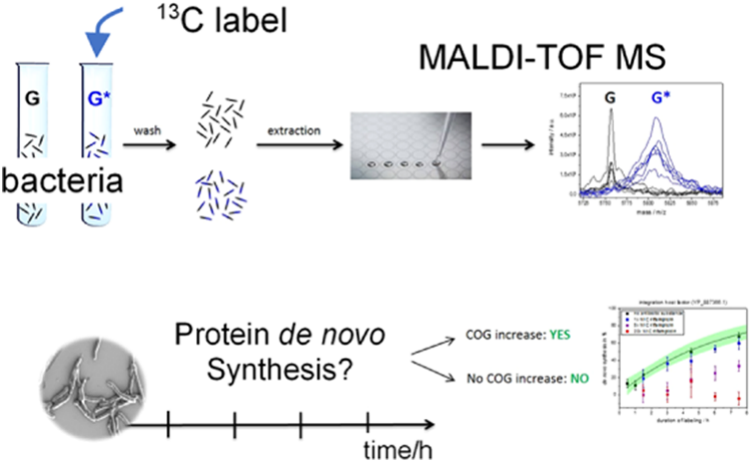

Understanding the metabolic pathways of mycobacteria is essential to identify novel antibiotics and to compose synergistic antibiotic regimens against tuberculosis, one of the world’s most deadly infectious diseases with >1.7 Mio yearly deaths. We present a novel proteomics approach for the dynamic measurement of the nascent fractions of specific proteins. We use nontargeted stable isotope incorporation to label the nascent proteins after adding glycerol-1,3-^13^C_2_. The analysis is performed using matrix-assisted laser desorption–ionization time-of-flight mass spectrometry (MALDI-TOF-MS) with a self-programmed script, allowing quantitative data. We compared the *de novo* synthesis of proteins under regular growth conditions and the effect of four antimicrobials, including rifampicin as a first-line drug, linezolid and bedaquiline as second-line drugs, and benzithiazinone-043 as promising drug candidates against tuberculosis. Changes in the synthesis of individual proteins, either due to antimicrobial action or due to regulations in the organism, can be followed in high temporal resolution within the 1/2 doubling cycle of mycobacteria. The analysis of *de novo* protein synthesis offers a fast screening and testing tool, allowing assessment of the onset and extent of antimycobacterial activity or regulatory phenotypes in different organisms. Due to the untargeted approach, it can be used in model strains and clinical isolates alike and does not require genetic modifications. The dynamic readout and labeling reveal the onset of action of drugs or drug candidates and allow for the prediction of synergistic effects of several substances.

## Introduction

Tuberculosis (TB) caused by *Mycobacterium tuberculosis* complex is one of the most deadly human infectious diseases, claiming more than 1.7 Mio lives each year.^1^ Although the disease is generally curable, treatment success is hampered by the necessity of a prolonged (4– ≥ 12 months) and side effect-prone treatment.^2^ Despite sustained efforts to develop new therapeutic options and better diagnostic tools, *M. tuberculosis* remains a WHO priority organism and a worldwide public health concern. There is an urgent need to find better diagnostic as well as therapeutic protocols to provide better treatments.^3^ Current methods to rapidly find and assess the effects of potential antimicrobials mainly rely on GFP production in genetically modified model organisms, resazurin color change, or the reduction of colony-forming units (CFU) after certain exposition times.^4−6^ Alternatively, two-dimensional gel electrophoresis and shotgun proteomics have been used to describe the protein composition of the bacterial population under treatment to measure treatment efficiency.^7−11^ In 2021, Bendre et al. gave insights into membrane composition and organization of mycobacterial membrane proteins, highlighting the importance of deciphering the modes of action of these proteins for the discovery and development of novel therapeutics.^12^ Protein synthesis can be used as an excellent indicator to monitor the metabolic viability of organisms, as this is a central step where multiple vital functions of the organism interplay. Further, almost all antimycobacterial treatments eventually directly or indirectly impair protein synthesis. Compensatory reactions of the organism to adapt and escape the actions of antimicrobials will manifest in a change in the production rates of certain proteins. However, as currently used approaches only address the steady-state levels of proteins and not the *de novo* protein synthesis, neither quick regulation phenotypes nor the termination of protein production can be measured. Despite the obvious importance of studying dynamic changes in the proteome of mycobacteria under treatment, well-defined protocols are missing.^7^

Metabolic labeling with stable isotopes has been applied in different organisms earlier, exchanging the carbon source for ^13^C.^13^ The average protein dynamics has been studied by determining the total ^13^C content in protein extracts after labeling. Stable isotope labeling by amino acids in cell culture (SILAC) has been used for sequence-specific information but requires specific amino acid-synthesis-deficient mutants.^14^ However, mycobacteria cannot easily be made auxotrophic by certain amino acids. Thus, SILAC has not been used in mycobacteria so far. Transcriptional analyses were used to address changes in the gene expression of mycobacteria. However, as many drugs target the protein synthesis machinery directly rather than RNA polymerases, the observed transcriptional changes are likely to be indirect effects and do not necessarily reflect the protein function. Furthermore, none of the commonly used techniques allow for the determination of the nascent protein fractions.

In this study, we performed nontargeted stable isotopic labeling of mycobacteria in rich media, subsequent protein extraction, and matrix-assisted laser desorption–ionization time-of-flight mass spectrometry (MALDI-TOF MS) readout (see Figure S1). We demonstrate the possibility of extracting quantitative information from MALDI-TOF spectra by introducing a novel method of data analysis. Using the center of gravity (COG) around “the *m*/*z* position of a protein isotopolog cluster upon adding ^13^C-labeled glycerol (G*), we observed rapid labeling of most proteins, resulting in shifts of mass signals to higher *m*/*z* values. Thereby, the mass shift of full-length protein signals can be followed until they reach a saturation level. Extracting the labeling kinetics of specific proteins from the temporal evolution of their mass signatures enables the parallel determination of the nascent protein fraction for many specific proteins. This allows, for instance, the assessment of protein turnover rates, also under steady-state conditions, and the sensitive detection of changes in protein production rates reflecting regulation. Such information is not easily accessible with current techniques, as these cannot differentiate old from newly translated mycobacterial proteins. Assessing the changes in nascent protein fractions would allow for not only quickly detecting bacterial death but also all kinds of regulatory changes caused by potentially lethal or nonlethal novel drugs or drug candidates.

The presented analysis of *de novo* synthesized proteins offers a fast (within 1/2 doubling cycle) screening and testing tool to assess the onset and extent of anti-TB drugs in subinhibitory and inhibitory concentrations and different organisms or regulatory phenotypes. Due to the untargeted approach, it can be used in model strains and clinical isolates alike and does not require genetic modifications. The dynamic readout and labeling reveal the onset of action of drugs or drug candidates and enable the prediction of synergistic effects of several substances.

## Experimental Section

### Bacterial Strains and Growth Conditions

Mycobacterial strains used in this study were *Mycobacterium smegmatis* mc^2^155 wild type (ATCC 700084) and *Mycobacterium* regarding culture conditions and MIC determination, see the Supporting Information (Chapter S2 and Figure S2). For *tuberculosis* H37rv (ATCC 25618), the MIC determination was performed in an identical way; the values are given below. For details of the isotopic labeling, the cultivation medium was enriched with 2.41 μL of glycerol-1,3-^13^C_2_ per mL of medium (Sigma-Aldrich).

### Sample Preparation

Sample preparation, extraction, and identification were performed as outlined in the Supporting Information, Chapter S1.

### Mass Spectrometry

#### MALDI-TOF MS Analysis

Each 1.4 μL of the protein extract was spotted directly onto a polished steel MALDI target plate, dried at 37 °C, and overlaid with each 1 μL of MALDI matrix (α-HCCA [10 mg/mL α-cyano-4-hydroxy-cinnamic acid–50% acetonitrile–2.5% trifluoroacetic acid]) (Bruker Daltonik, Bremen, Germany). MALDI-TOF MS measurements of the dried spots were performed with a Microflex LT/SH benchtop mass spectrometer (Bruker Daltonik GmbH, Bremen, Germany) equipped with a 60-Hz nitrogen laser controlled by FlexControl software (version 3.4.135.0, Bruker Daltonik GmbH, Bremen, Germany). Spectra were recorded in positive linear mode. The parameter settings were optimized for the mass range between 2000 and 20,000 Da and were as follows: ion source 1:0 kV, ion source 2:18.25 kV, pulsed ion extraction time: 130 ns. Gain and laser power were set to the manufacturer’s recommended values for bacterial identification. An external standard was chosen for instrument calibration, containing masses between 3637.8 and 16,952.3 Da (bacterial standard, BTS, Bruker Daltonik GmbH, Bremen, Germany). All acquisitions were recorded automatically in the instrument software and based on averaging 240 satisfactory pulses in 40 pulse steps (MBT_AutoX).

#### LC-MS Measurements for Collecting Protein Fractions

Proteins were analyzed by an HPLC system (Thermo Fisher Scientific) connected to an Orbitrap *Q exactive* mass spectrometer (Thermo Fisher Scientific). The protein separation was done with a C8 column (Agilent, 2.1 × 150 mm^2^ with 3.5 μm beads) at 30 °C; the flow rate was 0.3 mL/min; and the injection volume was 20 μL. A gradient solvent program was used, where acetonitrile and water (both containing 0.1% TFA) were used as the mobile phase. The initial composition of 5% acetonitrile was increased to 10% within 3 min, followed by increasing to 50% within 87 min and to 95% in 10 min. After 10 min of constant composition, the acetonitrile was linearly decreased to 5% in 1 s and held for 10 min. The mass spectrometer was operated in positive ionization mode at an ultrahigh resolution of 50,000 (2 Hz), 3 microscans, and a maximum injection time of 250 ms. Lock masses were set at 279.1591 and 391.2842. During the measurements, the nebulization gas was set at 40 L/h, and the auxiliary gas was set at 5 L/h. Spray voltage was set at 3.5 kV, capillary temperature to 350 °C, capillary voltage to 25.00 V, tube lens voltage to 175.00 V, and the skimmer voltage to 46.00 V. Fractions were collected every 30 s by placing a flow splitter (dividing the flow rate by half) directly in front of the sample inlet of the ESI. After MALDI-TOF verification of each collected fraction, the fractions (64, 77, 89, 93, 112, 117, 118, and 126) from two to four injections, including the proteins of interest (*m*/*z* 3160.89, *m*/*z* 4156.75, *m*/*z* 4554.42, *m*/*z* 5554.09, *m*/*z* 9541.26, *m*/*z* 4332.98, *m*/*z* 5771.84, see SI, Figure S6) were dried (SpeedVac vacuum concentrator, Thermo Fisher Scientific), digested, and identified by Nano-LC-MS/MS.

#### Nano-LC-MS/MS Analysis for Protein Identification

An aliquot of 5 μL of the digested protein sample was loaded directly onto an analytical C18-nanoRP column (120 × 0.075 mm^2^) and separated by a linear gradient from 2 to 40% acetonitrile (0.1% formic acid) in 30 min and online infused into an LTQ Orbitrap mass spectrometer (Thermo Fisher Scientific) for MS/MS analysis. The mass spectrometer was run in data-dependent detection, acquiring MS/MS spectra of up to 5 detected precursors in the ion trap while recording the accurate peptide mass in the orbitrap analyzer at a resolution of 30,000 (at *m*/*z* 400). MS/MS data were acquired in the ion trap analyzer, employing collision-induced dissociation at a normalized collision energy of 35% and an activation time of 30 ms.

### Data Processing and Statistical Analysis

#### Protein Identification

The resulting mass spectral data were searched against a combined forward/reversed database of *M. smegmatis* proteins (Uniprot 2021/01) database using the MaxQuant (2.1.0.0) software suite (www.maxquant.org by the Max-Planck Institute of Biochemistry, Martinsried, Germany). Methionine oxidation, protein N-terminal acetylation, and glutamine deamidation were allowed as variable modifications, with carbamidomethylation of cysteines as a fixed modification. Peptide and protein hits were filtered for 1% reversed hits and a minimal identification score of 10 for unmodified peptides and 25 for modified peptides. Due to the small size of the isolated proteins, protein hits with one identified peptide were accepted upon manual inspection.

#### MALDI-TOF MS Data Analysis

For data analysis, mainly [M + 2H]^2+^ MALDI-TOF peaks were analyzed except for *m*/*z* 9541.26 and *m*/*z* 5554.09, where the [M + H]^+^ was used for analysis. All MS data were converted to the mzXML format by the free software MSConvert and further evaluated by in-house-made scripts programmed in Matlab (Matlab 2020b, The Mathwork, Inc.). First, the background was fitted by a spline and removed using the Matlab command “msbackadj” with a window size as well as a step size of 200 data points. The next step was a Savitzky–Golay filtering (width 4). The “findpeaks” command identifies local maxima for peak detection. A minimum peak intensity of 150 counts, a peak prominence of 30, and a peak distance of 3 data points were used.

All peaks thus identified in a specific *m*/*z* range were summed up and weighed by their respective position relative to the *m*/*z* position of the nonshifted peak position, resulting in a center of gravity (COG) in direct analogy to the mechanical equivalent. The mass range was adapted individually for each component investigated. The shift of the COG reaches a specific maximum value, which represents the maximum number of carbon atoms contained in the respective molecule that can be replaced with a ^13^C atom. Not all C atoms can be replaced because an almost instantaneous incorporation of ^13^C into the amino acid pool for protein biosynthesis causes a certain relative composition of labeled and unlabeled precursor building blocks (see SI, Chapter S7).

The evaluation approach based on the COG was chosen because an exact description of the isotope distribution, particularly for the labeled organisms, is not possible. The COG approach is robust and suitable for any potential target molecule. To facilitate intuitive interpretation and comparison of the shift values, the COG values were normalized to the corresponding saturation values and given as the percent of the maximum shift. This maximum value was determined by fitting the following equation to the temporal behavior of the COG values
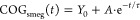
1with τ being the specific rise time, *Y*_0_ being the maximum offset, and *A* allowing for a normalization of the maximum saturation to 100%. As for *M. tuberculosis*, the rise times are significantly longer; they cannot be neglected and thus are accounted for by a temporal offset *t*_0_
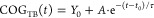
2

## Results and Discussion

### Choice of Labeling Substance

To perform a global, unspecific isotope labeling of proteins in mycobacteria in rich media, we made use of the fact that mycobacteria can utilize multiple different carbon sources in parallel.^15^^6^ Glycerol is converted to serine/glycine, alanine, and aspartate, as well as acetyl-CoA *via* phosphoglycerate, pyruvate, or oxalacetate, respectively. Further, glycerol ends up rapidly in the Acetyl-CoA pool, thus feeding into various metabolic pathways, including lipid synthesis and amino acid synthesis.^16^ Hence, glycerol-1,3-^13^C_2_ (G*) should lead to the unspecific, highly efficient, and rapid labeling of *de* novo synthesized proteins. Indeed, after the addition of G* to a fully supplemented growth medium, a rapid mass increase of almost all mass signals (∼90% in the range between *m*/*z* 2000 and *m*/*z* 12000 within 24 h) was detected. To gain appropriate G* concentrations in the medium, a range between 0.47 and 9.40 ng/mL was assessed (see the SI, Figure S3). A concentration of 2.7 ng/mL was chosen as it guarantees stable labeling over the complete duration of the experiment. As this concentration is way below the range that can be achieved within a mouse model (up to 28 μg/mL),^17,18^ it might, in the future, also be adaptable for *in vivo* experiments.

### Quantitative Data Analysis of MALDI-TOF Spectra

**Figure 1:**
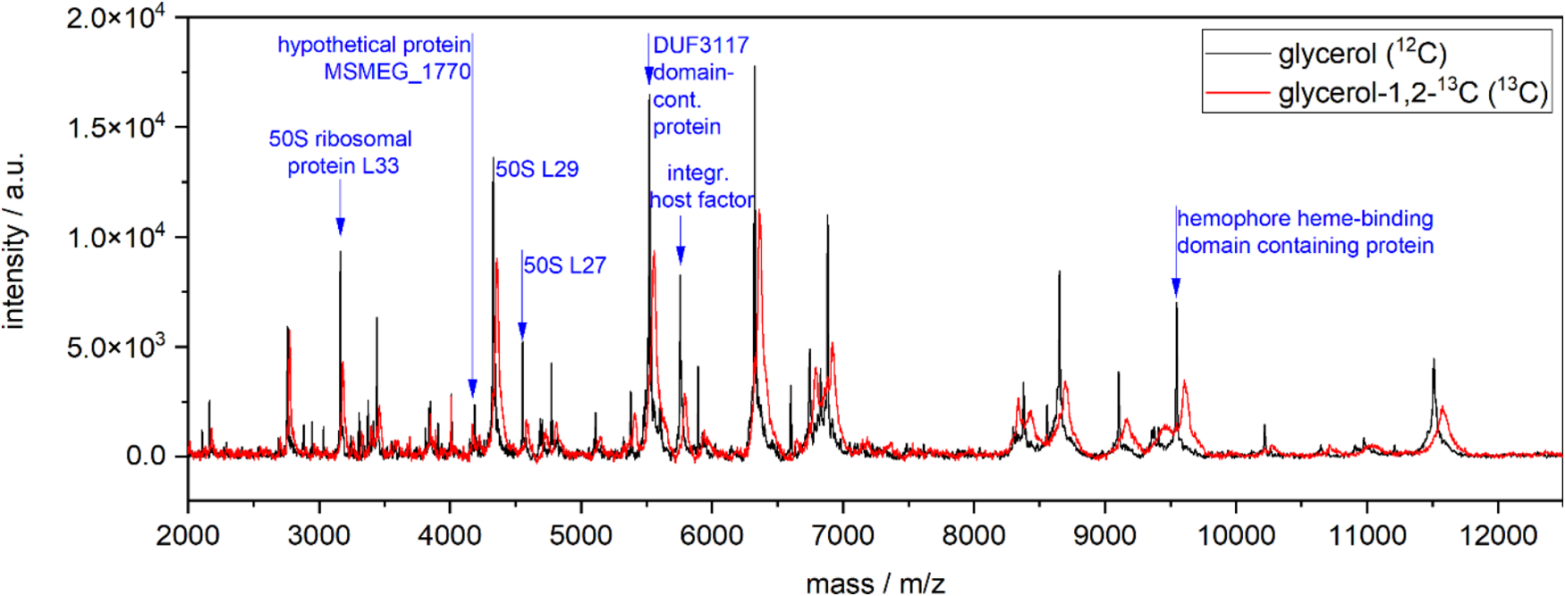
MALDI-TOF spectra of protein extracts of *M. smegmatis* cultures after 24 h of growth in either light (glycerol (^12^C), G, black) or heavy (glycerol-1,2-^13^C (^13^C), G*, red) medium.

**Figure 2:**
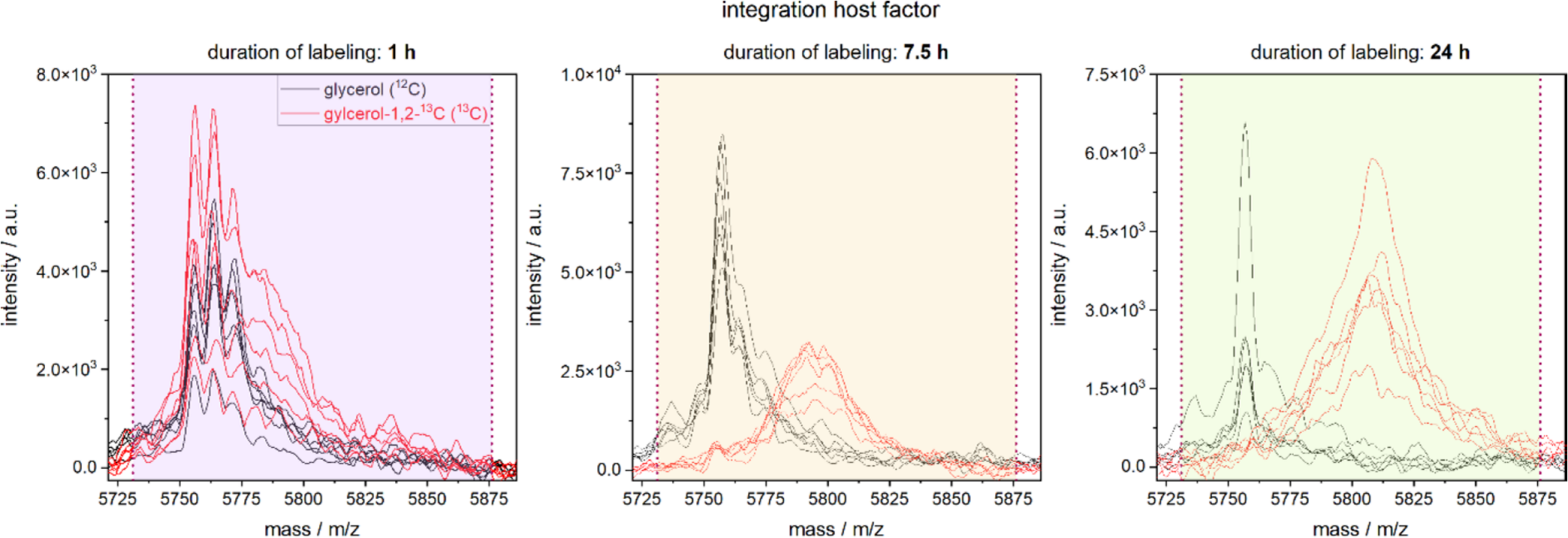
Following the COG for *M. smegmatis* bacteria cultured either in light (glycerol (^12^C), G, black) or heavy (glycerol-1,2-^13^C (^13^C), G*, red) medium over time (sampling time points: 1 h, 7.5 and 24 h) exemplified for *m*/*z* 5771.84, identified as integration host factor (A0QWS8).

With the widely distributed MALDI-TOF MS machines for routine analysis, the isotopic resolution of large proteins cannot be achieved, making the temporal evolution of mass shifts difficult. The complexity of insufficiently resolved spectra, in combination with fluctuating overall spectra intensities, aggravates the direct evaluation of mass shifts in MALDI-TOF mass spectra. However, analyzing the COG of all mass signals around the unlabeled mass signals was found to be highly reproducible and, by its nature, independent of the absolute intensity of the respective peaks (see Figure 2). The variability in COG determination was 5765.79 ± 2.09 for 1 h for G, 5771.49 ± 1.92 for 1 h for G*, 5760.14 ± 1.90 for 7.5 h for G, 5795.07 ± 1.76 for 7.5 h for G*, 5759.34 ± 3.11 for 24 h for G, and 5807.63 ± 1.31 for 24 h for G* (*n* ≥ 19 for each time point and condition). Limit-of-detection studies were performed to demonstrate the sensitivity of the newly established MALDI-TOF technique. Extracts of ≤102,000 bacteria cells per target spot were sufficient to produce multiple, consistent measurements. Amounts of >10^6^–10^7^ were also measured without problems or inhibition. For one spot on the MALDI-TOF target, 1 μL of the 20 μL eluate was used. Only a minute fraction is analyzed from each spot by the laser; thus, spectra such as those shown in Figures 1 and 2 are generated from spots containing extracts of way less than 102,000 cells.

**Figure 3:**
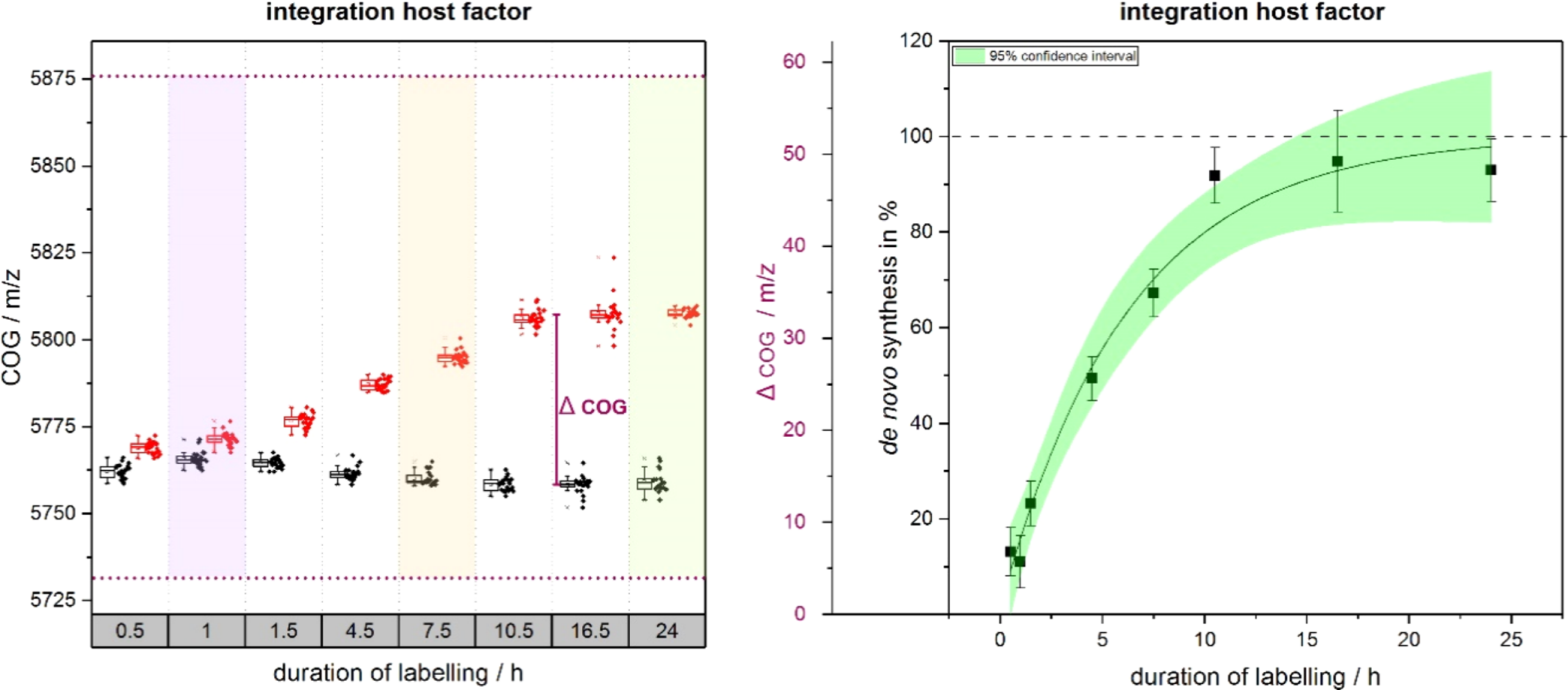
Typical results for individual assessment steps. Left side: the *de novo* synthesis calculation was performed evaluating the COG around the native protein mass; right side: derived de novo synthesis rate over time after normalization.

The COG values of the peaks from bacteria grown in G are subtracted from those grown in G* medium to determine a differential COG (ΔCOG) value (see Figure 3). This eliminates any possible artifacts due to mass changes of nature other than the incorporation of ^13^C into the product, possibly occurring within the analyzed mass range. Changed post-translational modifications (*e.g.*, formylation, methylation) or an altered milieu, leading to oxidation or salt adducts within the samples, do not change the ΔCOG, as they occur in both groups simultaneously.

After hours of labeling, we observed the saturation of the total mass shift. Thus, ΔCOG induced by isotope feeding can be normalized between 0 (no contribution, unlabeled culture) and 100% (full contribution, saturated total mass shift). By using these normalized ΔCOG values, relative quantification of the mass shift of the individual analyte population can be achieved, based only on the fair assumption that ^13^C enriched proteins of the same amino acid sequence are ionized as well as their unlabeled counterparts within the precision of our measurements. This allows for quantitative measurements despite the fact that the use of MALDI-TOF-MS for absolute quantification is not generally accepted.^15^ The ΔCOG calculation remains reliable for low concentrations as long as at least one peak per specific analyte is accessible in the mass spectrum of the labeled as well as the corresponding nonlabeled group. The approach allows for measurement over a wide range of protein sample concentrations without requiring further dilutions, creating a very robust analytical tool. The accuracy of the relative mass shifts measured in COG was confirmed using HPLC-coupled to ESI-Orbitrap analysis (see SI, Figure S5). Furthermore, with this comparison, we could show charge states of mainly +2 and partly +1 and +3 for all investigated proteins. This is in agreement with the literature where Choi et al. investigate charge states of cytochrome c (∼12.000 Da) using HCCA as a matrix.^19^

### Identification of Proteins

In this study, we follow the *de novo* synthesized fractions of seven proteins chosen as analytes with measured *m*/*z* values between 3160.89 and 9541.26. Those seven were selected as they were easily accessible in the spectra, were not overlaid by other proteins, and showed the highest intensities. These specific proteins were isolated using HPLC-MS in combination with a flow splitter directly in front of the ESI; each fraction was verified using MALDI-TOF MS (see SI, Figure S6), and corresponding fractions were enzymatically digested and identified using nano-LC-MS/MS in combination with MaxQuant analysis (Table 1 and SI, file “Protein Identification_F.xlsx”).

**Table 1:** Main *m/z* Peak Correlated to the Identification Obtained by Enzymatic Digestion, LC-MS/MS Measurement, and MaxQuant Analysis

*m/z* peak MALDI	3160.89 [M + 2H]^2+^	4156.75 [M + 2H]^2+^	4332.98 [M + 2H]^2+^	4554.42 [M + 2H]^2+^	5554.09 [M + H]^+^	5771.84 [M + 2H]^2+^	9541.26 [M + H]^+^
identified protein	50S ribosomal protein L33	hypothetical protein MSMEG_1770	50S ribosomal protein L29	50S ribosomal protein L27	DUF3117 domain-containing protein	integration host factor	hemophore heme-binding domain-containing protein
primary accession	A0A1A3PVM2	A0QTA4	A0QSD9	A0R150	A0R2E3	A0QWS8	A0QP20
gene/ORf name	rpmG	MSMEG_1770	rpmC	rpmA	MSMEG_5081	MSMEG_3050	MSMEG_243

The seven proteins we evaluated comprise three 50S ribosomal proteins, L33, L29, and L27, which play an important role in mycobacterial membrane processes and, obviously, the ribosome. Mycobacterial integration host factor (mIHF) is one of mycobacteria’s few identified nucleoid-associated proteins (NAPs) so far. These proteins play an essential role in chromosome condensation and organization.^20^ Hemophore, hemo-binding (MHB) is a coiled-coil molecule that binds free hemo in mycobacterial cytoplasm to deliver it to membrane proteins for shuttling through the membrane. The biological role of DUF3117 and the hypothetical protein MSMEG_1770 is currently unknown. Those proteins allow for the investigation of different regulatory features of *M. smegmatis* within this study.

### Turnover of Mature Mycobacterial Proteins

We followed the COG shift of seven representative proteins for 24 h, and surprisingly, the labeling effect saturated in a limited time period (see SI, Figure S7). After 1.5 to 10.5 h, no further significant mass changes of the products occurred while the bacteria were still in rapid growth, as seen by an increase in OD_600_. This demonstrates that the labeling saturates due to reaching the maximum contribution obtainable in the model and not by changes in metabolism or growth of the bacterial culture. We conclude that within the chosen conditions, ^13^C from G* is incorporated into the current protein biosynthesis. Thus, mature proteins that were synthesized *de novo* after adding G* are labeled in a highly reproducible and robust manner *via* one or several pathways. The relevant reactions thereby saturate fast; other, slower pathways, which would lead to a prolonged *m*/*z* shift, are negligible (for further explanation, see SI, Chapter S7 and Figure S7).

We observed that during regular growth, the ΔCOG values of the proteins investigated follow an inverse exponential behavior over time with distinct saturation values and time constants (see Table 2 and SI, Figure S8). Time constants and saturation levels were found to be reproducible and stable for each individual investigated protein in repetitive experiments. Calculated *de novo* synthesis rates of structural proteins, such as ribosomal proteins, are expected to be within the observed duplication rate of the organism because it is known that ribosomal biosynthesis follows the growth of the organism under exponential growth conditions.^21^ In fact, this was confirmed for structural proteins L27, L29, and L33. This observation confirms the hypothesis that the incorporation rate of ^13^C can be used to determine the fraction of each specific nascent protein prevalent in the organism at the analyzed time point.

**Table 2:** Time to Reach 50% Contribution (Calculated Assuming Exponential Behavior) and the Percentage of ^13^C Atoms to the Total Number of C Atoms for the Chosen Seven Proteins

identified protein	time to reach 50% contribution/h	percentage of labeled C atoms (%)
integration host factor	4.35 ± 0.92	20.9
hemophore heme-binding domain-containing protein	3.48 ± 0.60	18.6
hypothetical protein MSMEG_1770	1.50 ± 0.62	13.2
DUF3117 domain-containing protein	4.56 ± 1.46	11.0
50S ribosomal protein L27	4.72 ± 1.15	26.5
50S ribosomal protein L29	5.65 ± 2.06	13.7
50S ribosomal protein L33	5.30 ± 0.99	17.9

The average labeling efficiency of the investigated proteins was found to vary between 11% and 27% of all carbon atoms. This variation is most likely caused by the different amino acid compositions of the different proteins. The only labeling substance used in the experiment is glycerol-1,2-^13^C, while no free amino acids are provided in the media, resulting in all proteins being synthesized either from recycled proteins and their breakdown products or by *de novo* synthesized amino acids as building blocks.

### *De Novo* Protein Synthesis Analysis Reveals the Early Onset of Antibiotic Actions

Using this newly developed method to investigate the action of antimicrobial substances, we measured the dynamics of protein *de novo* synthesis of seven identified proteins upon application of different antimicrobial substances featuring completely different modes of action. BTZ inhibits the cell wall synthesis,^22^ LZD blocks the translation on the ribosomal level,^23^ and BDQ inhibits the ATP synthesis,^24,25^ while RIF blocks the DNA-dependent RNA synthesis.^26^ Multiples of the minimal inhibitory concentrations (MIC, corresponding to IC_90_) for each substance were used in the further experiments (1×, 5×, 20× MIC).

**Figure 4:**
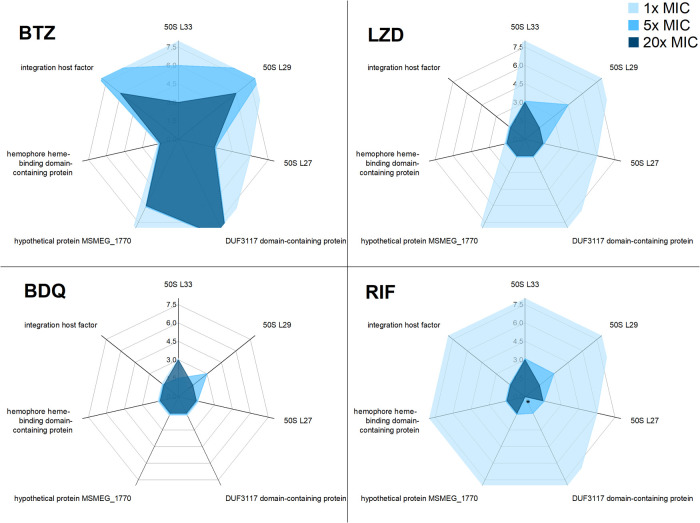
Spider diagram of the time to first significant (for explanation, see SI, Chapter S8) change from wild-type level production of the seven proteins chosen for analysis at four different antibiotics (BTZ, LZD, BDQ, and RIF), each at three different concentrations (1×, 5×, and 20× MIC).

In Figure 4, the time of the first significant changes in ΔCOG as compared to the wild type is plotted, with increasing times further out of the center of each spider diagram. In brief, a small blue spot signifies a fast reaction, each leg of the spider representing one protein, while each spider stands for one antibiotic. Changes in ΔCOG are considered significant when the error bar of a measuring point (corresponding to one standard deviation 1 × *s*, calculated with error propagation) does not overlap with the confidence interval of the curve fit.

This plot immediately reveals that BDQ, which terminates the ATP production rapidly, leads to the cessation of protein biosynthesis within 1.5 h, t. CFU numbers remain virtually unchanged (no significant change as compared to the control group; P-value between 0.17 and 0.85 at a significance level of 0.05; see SI, Figure S10) because cells are not dead yet. In contrast, the transcriptional inhibitor RIF 1× MIC shows no significant effect in the first 6 h period. Upon exposure to 5× MIC, protein *de novo* synthesis is reduced as early as 1.5 to 3 h due to the short half-life of mRNA, which is no longer produced. Again, at this time point, no significant reduction of CFU is observed (see SI, Figure S10). LZD, which blocks protein biosynthesis directly, does not significantly affect the shift of structural proteins (L33, L29, or L27) at 1× MIC for 6 h, whereas *de novo* production of integration host factor and hemophore hemo-binding domain-containing protein is reduced as early as 1.5 h. This shows apparently incomplete ribosomal blockage and time for LZD to reach its target. Finally, BTZ, which blocks DprE1, required for the cell wall synthesis leads to almost unchanged protein biosynthesis of the structural proteins L33 and L29 as well as on the DUF3117 domain-containing protein for a long time. Again, hemophore hemoprotein-binding domain-containing protein *de novo* synthesis is reduced significantly after 1.5 h even upon 1× MIC, which confirms the sensitive detection of individual protein production rate changes caused by regulation. In the BTZ group, CFU numbers are even raising slightly in that time frame (see SI, Figure S10). During the same time, optical density-based techniques did not demonstrate a significant change either. Commonly used screening techniques for antibiotics are, *e.g.*, resazurin color change and the reduction of colony-forming units (CFU) assays.

### Antibiotic Effects over Time/Dynamics

Antibiotic effects (1×, 5×, 20× MIC) were observed over a period of 13.5 h using staggered timelines with different labeling starts (the addition of G* after 0, 3, and 6 h with 7.5 h labeling duration each; for further information, see SI, Figure S11). The staggered timelines are required, as saturated ΔCOGs do not allow the detection of changes in production rates anymore, thus limiting the meaningful observation time. To overcome this limitation, the addition of G* can be delayed to start the labeling process later and thus detect alterations in production rates at later time points while maintaining high sensitivity for changes. In the following studies, delays of 3 and 6 h were chosen.

**Figure 5:**
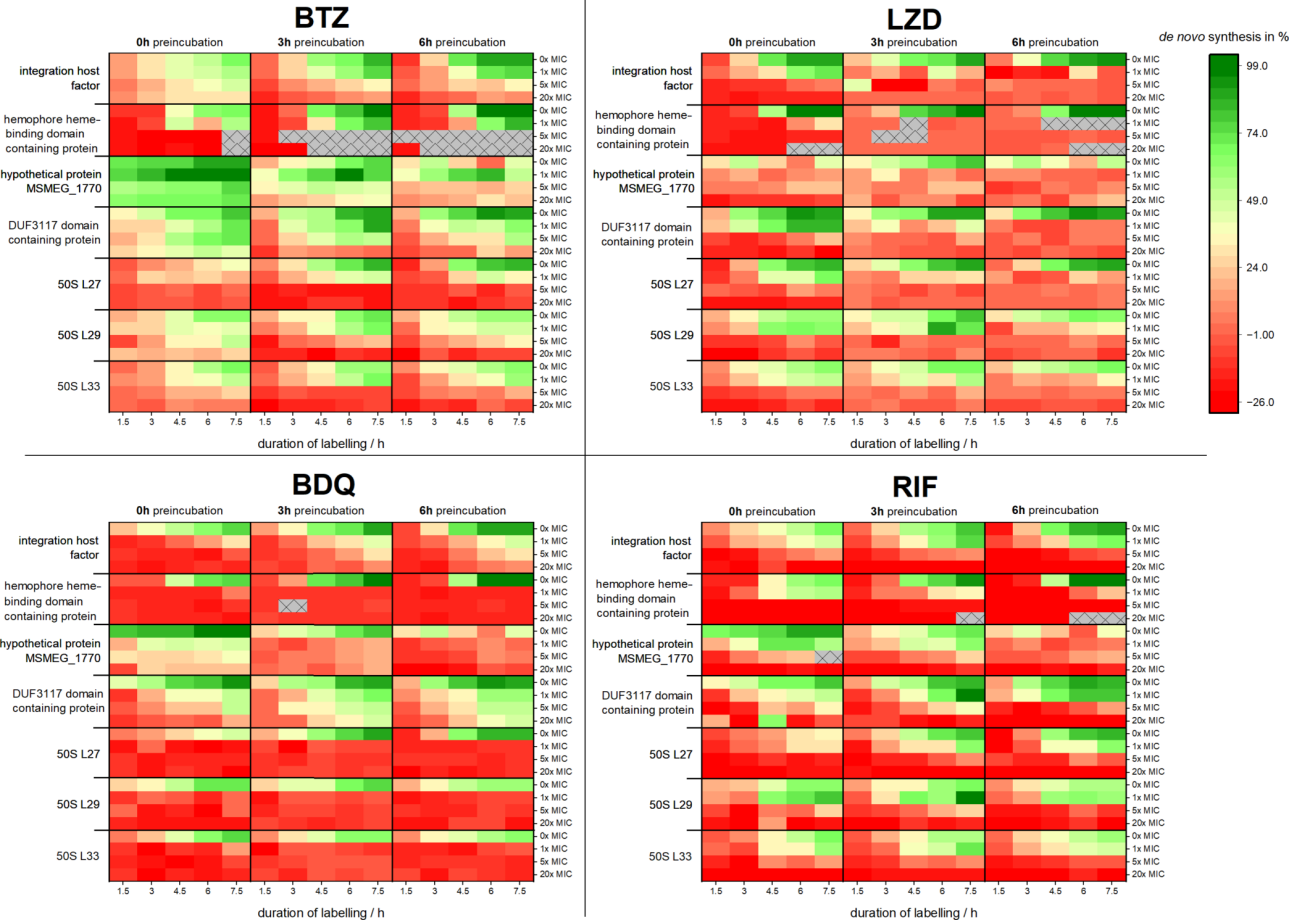
Heat map diagram of the normalized ΔCOG values over time. Each of the four matrices displays the normalized ΔCOG for four different antimicrobials (BTZ, LZD; BDQ, and RIF) of the investigated proteins (seven horizontal blocks) for different time points (vertical columns; 1.5, 3, 4.5, 6, and 7.5 h after the addition of the tracer G*). Different antibiotic preincubation times (vertical blocks; 0, 3 h, and 6 h) were used and are represented by the three columns separated by the black line. Dark green: 100% contribution/nascent, and dark red: 0% contribution/synthesized before labeling.

We observed a significant upregulation of the production of hypothetical protein MSMEG_1770 protein within hours of confrontation with 1× MIC BTZ. At this time point, no decline in CFU is observed. In contrast, within the seven exemplarily presented proteins, no upregulation above the wild-type controls is observed in the BDQ group. However, there is substantial residual protein synthesis of DUF3117 domain-containing protein and integration host factor after more than 9 h of confrontation, even with 20× MIC of BDQ. This is surprising, as significant changes in protein biosynthesis can be appreciated very early on. The drastic drop in protein *de novo* synthesis is explained by the mode of action of BDQ, causing a decrease in intracellular ATP levels. After 9 h of BDQ 20× MIC, the reduction of CFU as compared to the start culture is about 1.2 ± 0.17 orders of magnitude. This demonstrates that the surviving cells produce only a small number of proteins. Thereby, especially ribosomal structural protein biosynthesis (L27, L29, and L33 analyzed here) is almost completely abrogated even after 1× MIC for only 1.5 h (deep red in the heat map Figure 5), thus underlining the technique’s sensitivity to pick up the specific signal of the small fraction of surviving cells within the mixture.

With LZD, the time frame of regulation of DUF3117 domain-containing protein can be exemplarily visualized. Its production at 1× MIC LZD reaches saturation with a value of 71.4 ± 11.5% contribution at about 4.5 h and thus is produced significantly more than other proteins, *e.g.*, hemophore hemo-binding domain-containing protein (0.9 ± 1.8%) or integration host factor (21.9 ± 9.4%) under this condition. Staggered timelines reveal that 7.5 h after 1× MIC exposure. However, the *de novo* synthesis of DUF3117 domain-containing protein has stopped. At this time, CFU reduction is barely significant, with about 70% of cells still being viable. It thus suggests that the bacterial cells have downregulated the production of the DUF3117 domain-containing protein at that point in time. The complete cessation of protein *de novo* synthesis is an interesting marker for achieving a severe effect on the bacterial organism. We see that 20× MIC of LZD leads to a protein synthesis stop after 7.5 h in all presented proteins, while a reduction of bacterial load of 2.2 ± 0.47 orders of magnitude is reached. In contrast, BTZ 20× MIC only leads to the cessation of hemophore hemo-binding domain-containing protein production, while the other proteins remain in production, with DUF3117 domain-containing protein production not significantly impaired. At the same time, the reduction rate under this condition is less than 1 order of magnitude. RIF shows dose-dependent impairment of protein synthesis, with the 1 x MIC being quite inefficient. At 20× MIC, a fast reduction is seen; however, there is significant production of L29 after more than 7.5 h. This is surprising, as the supposed short mRNA half-lives and assumed complete suppression of DNA-dependent mRNA synthesis by RIF should not allow the organism to synthesize this protein. However, there is a significant number of surviving bacteria (10–20% of the starting population) at that time as well, obviously functional enough for protein synthesis.

### Antibiotic Effect on *M. tuberculosis*

**Figure 6:**
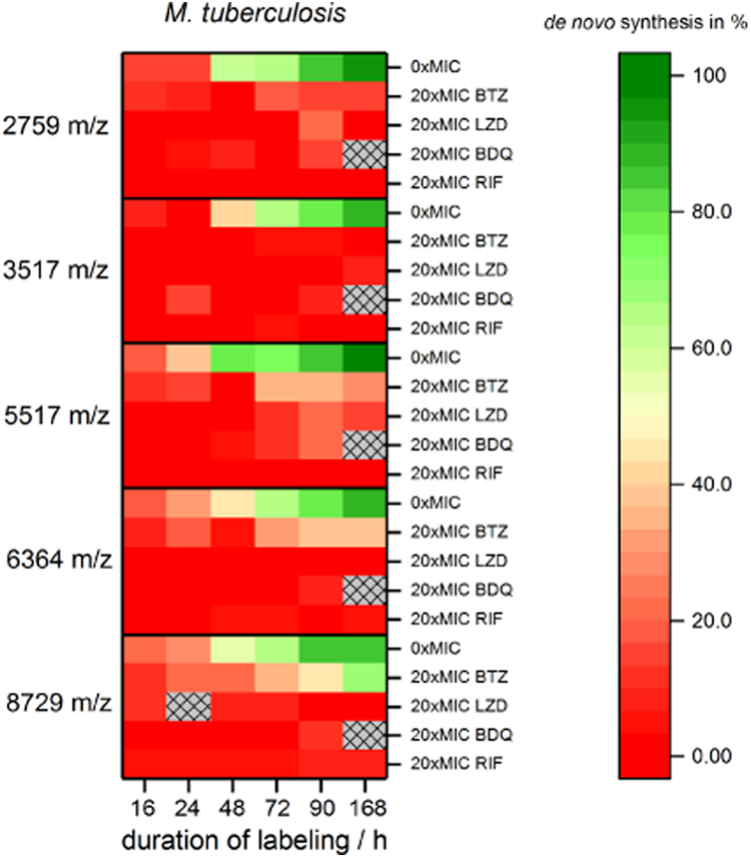
Values in the matrix indicate the ΔCOG of five chosen *m*/*z* values for different time points (16, 24, 48, 72, 90, and 168 h) during incubation with four different antibiotics (BDQ, BTZ, LZD, RIF). Dark green indicates unaffected protein synthesis (untreated control: 0× MIC), whereas dark red indicates the stop in protein *de novo* synthesis.

After using the techniques in the model organism *M. smegmatis*, we investigated *M. tuberculosis* complex strain H37Rv as confirmation. After the determination of the MIC values, which was performed in the same way as for *M. smegmatis* (see Supporting Information, Chapter S2), the dynamics of *de novo* production was observed for five analytes over 168 h. This duration corresponds to ∼7 duplication rates of H37Rv under the chosen conditions. Cultures were investigated without the addition of antimicrobial substances (0× MIC) and with 20× MIC of each antibiotic investigated in this study (BTZ, MIC 6.31 ng/mL; LZD, 1.32 μg/mL; BDQ, 0.14 μg/mL; RIF 0.014 μg/mL). During regular growth, the ΔCOG values followed the same inverse exponential behavior over time, as observed for *M. smegmatis* (see SI, Figure S4). However, the labeling onset in *M. tuberculosis* is delayed compared to *M. smegmatis*. The uptake of glycerol into *M. tuberculosis* cells is slower due to a lower number of available carbon transporters into the organism.^27^ Thus, the onset of labeling after glycerol uptake is no longer below the measurement accuracy for time points as for *M. smegmatis* and is thus accounted for when fitting the COG shifts. In *M. tuberculosis*, the first significant changes from wild-type level production in treated cultures using only five analytes were detected as early as 16 h, which is about half the duplication rate of the organism with roughly 24 h. For example, after 16 h, the labeling contribution for mass *m/*z 8729 is reduced from 20.1 ± 4.8 to 3.9 ± 1.8% and for *m*/*z* 6364 from 18.6 ± 6.9 to 1.9 ± 6.4% when incubated with 20× MIC RIF (Figure 6 and SI, Figures S12 and S13). The other antimicrobials also show drastic alterations very early on (Figure 6). As for *M. smegmatis*, the onset of antibiotic action can be detected within less than one duplication time for *M. tuberculosis*, demonstrating the potential of the technique for pathogenic mycobacterial isolates. It can be expected that with the investigation of many more different analytes, the speed for detecting different phenotypes will be further increased.

## Conclusions

Here, we describe a novel proteomics tool that augments existing protocols with a dynamic dimension, allowing the measurement of the nascent protein fraction in mycobacteria. The technique relies on MALDI-TOF-MS and untargeted stable isotope labeling. Labeling with stable ^13^C as a carbon source has been performed in multiple organisms and is a common technique for studying metabolic activity. The protocol described within this paper can be directly applied to clinical isolates and to prototype strains alike. Further, using a simple sample preparation and rapid measurements, the approach is scalable, allowing a fast, low-cost possibility to track the modes of action of different drugs and candidates on mycobacterial strains. For the analysis, neither genetic modification nor specific growth media are required. The concentrations of labeled tracers used to achieve labeling responses are low, allowing for cost-effective, large-volume experiments. We use ^13^C-labeled glycerol in a concentration of 2.7 ng/mL as a tracer substance. The concentrations needed have been described to be reached in mouse plasma in experiments, suggesting the possibility of using the technique also in animal models. The sample preparation and analysis pipeline is streamlined and fast. Extraction is performed using 96 well filter bottom plates (<1 h), followed by ultrarapid measurement (96 samples <1 h) in a MALDI-TOF MS. MS Data is evaluated based on automated center of gravity (COG) analysis, allowing for the extraction of quantitative information from MALDI-TOF mass spectra. This method was found to be highly reproducible and, by its nature, independent of the signal intensities. Thus, this technique is principally applicable to all MALDI-TOF MS spectra of organisms and allows for highly parallelized protein turnover measurements.

Tracking these mature protein signatures over time, the ΔCOG values follow exponential behavior with distinct saturation values and time constants. We could detect the action of the tested antibiotics within about half the regular doubling cycle for *M. smegmatis* (<1.5 h) as well as for *M. tuberculosis* (<16 h). This highlights the potential of the technique for ultrarapid screening for potential drugs and drug candidates, as well as potential uses in antimicrobial susceptibility tests (AST).^28^ Investigating in more detail the changes in the *dynamic translatome*, we demonstrate its capabilities with only a small fraction of the total proteins. We show the detection of regulatory phenotypes (up- and downregulation) as well as the cessation of protein biosynthesis.

With the generation of a mass spectrometry library comprising many more specifically identified proteins and metabolites, the presented workflow will allow further in-depth insight into the regulation of bacterial metabolism. Comparing the findings with HPLC-coupled ESI-Orbitrap measurements, we also demonstrate the feasibility of the automated COG approach for more elaborate MS techniques. This potentially broadens the number of analytes, which can be assessed in parallel while increasing the turnaround time and instrument/running costs. However, one might envisage performing rapid parallelized testing using fast and parallel analytics such as MALDI-TOF MS for screening and other purposes while obtaining much deeper dynamic information for certain time points or conditions using chromatography-ESI coupled protocols.

The mycobacterial *dynamic translatome* may prove to be a momentous method for understanding the mode of action and dynamics of antimycobacterial and may also help in resistance testing and rational drug design in the future.

## Supporting Information Available

Details of the experimental methods; determination of the minimal inhibitory concentration (MIC90) values; reasoning on 1,3-^13^C labeling by glycerol; a comparison of MALDI analyses with HPLC-ESI-MS analyses; discussion on the detection of the onset of antibiotic effects; and the results of the study on *M. tuberculosis* (PDF)
Protein identification (XLSX)
