# Supplementary material for: Dynamic Measurement of Protein Translation in Mycobacteria Using Nontargeted Stable Isotope Labeling in Combination with MALDI-TOF Mass Spectrometry-Based Readout
Source: Anal Chem. 2025 Feb 25;97(9):4850–9. doi: 10.1021/acs.analchem.4c03931 (PMC11912119; doi:10.1021/acs.analchem.4c03931)
Supplement: Supplementary file 1 — ac4c03931_si_001.pdf [file ac4c03931_si_001.pdf]

Supporting Information

Dynamic measurement of protein translation in mycobacteria using non-targeted stable isotope labeling in combination with MALDI-TOF mass spectrometry-based readout

Christoph Haisch[1,#,*], Anna-Cathrine Neumann-Cip[2,3,4,#], Axel Imhof[5], Andreas Schmidt[5], Ignasi Forne[5], Michael Hoelscher[2,3], Andreas Wieser[2,3,4,*]

1: Chair of Analytical Chemistry, Technical University of Munich, 85748 Garching, Germany

2: Division of Infectious Diseases and Tropical Medicine, LMU University Hospital, LMU Munich, 80802 Munich, Germany

3: German Center for Infection Research (DZIF), Partner Site Munich, 80802 Munich, Germany

4: Chair of Medical Microbiology and Hospital Epidemiology, Max von Pettenkofer Institute, Faculty of Medicine, LMU Munich, 80802 Munich, Germany

5: Biomedical Center, Protein Analysis Unit, Faculty of Medicine, Ludwig-Maximilians University Munich, 82152 Planegg-Martinsried, Germany

# shared first authors

***Corresponding authors**
Christoph Haisch
Lichtenbergstraße 4
85748 Garching
Germany
Haisch@tum.de
+49 89 289-154 504

Andreas Wieser
Elisabeth Winterhalter Weg 6
81377 Munich
Germany
andreas.wieser@lmu.de
+49 89 2180 78 296

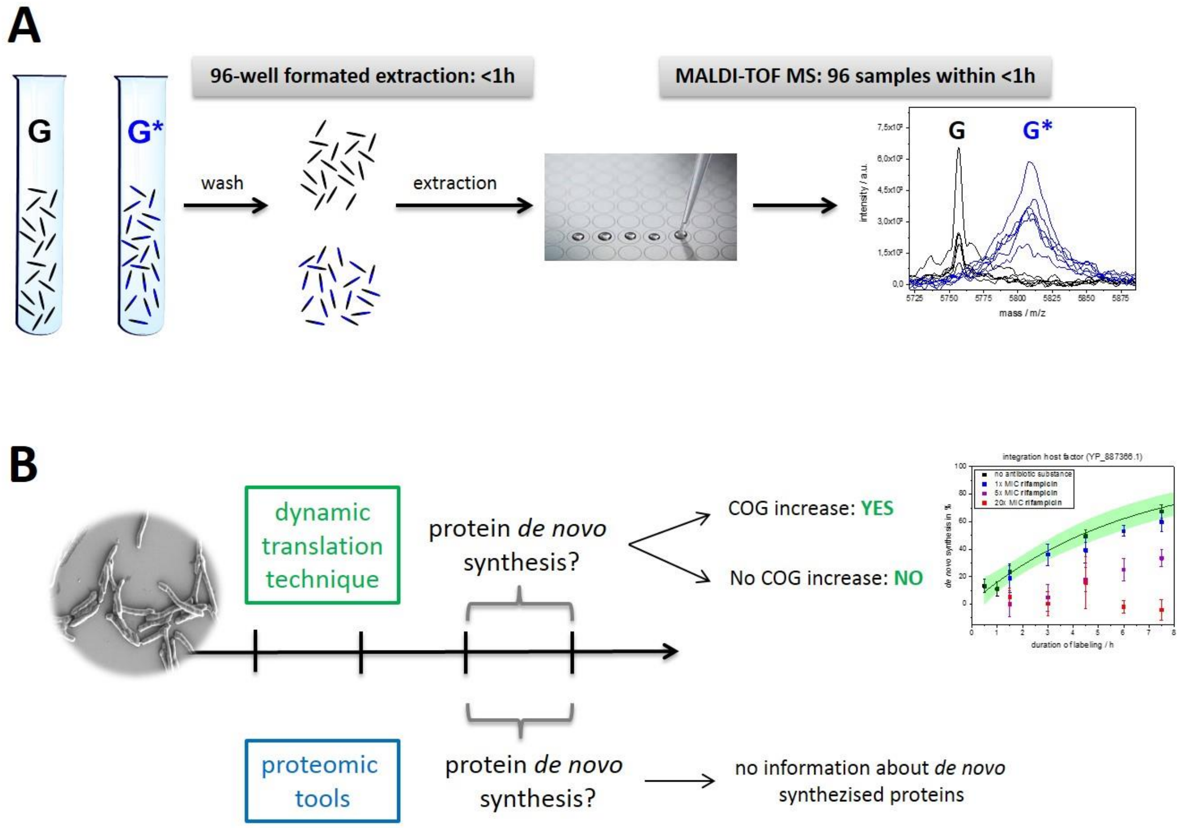

Figure S1: **A** Workflow of the fast, low-cost, and highly parallelized Matrix-Assisted Laser Desorption /Ionisation Time-of-Flight (MALDI-TOF) mass spectrometric method based on stable isotopic labelling to access the protein turnover of mycobacteria. **B** Difference between the newly presented dynamic translatome analysis and commonly used proteomic tools.

1 SI Experimental Methods

Bacterial strains and growth conditions

Culture Conditions: Frozen glycerol stocks of *M. smegmatis* (-80 °C, Middlebrook 7H9, OADC, 0.05% Tween-80, 20% glycerol) were thawed and initially cultivated at 37°C, 140 rpm for 15 h in cultivation medium (Middlebrook 7H9, OADC, 0.05% Tween-80). All experiments were conducted using a starting optical density at 600 nm (OD600) of 0.1 in fresh cultivation medium (centrifugation for 10 min at room temperature and 10,000 rpm followed by pellet resuspension in fresh cultivation medium). Antibiotic treatment was done with four drugs (BTZ043, HAPILA GmbH, Germany, linezolid, Sigma Aldrich, Germany, rifampicin, Sigma Aldrich, Germany, bedaquiline fumarat, MedChemExpress, Sweden) at different minimal inhibitory concentrations (1×, 5×, 20× MIC) and durations (0.5 to 24 h). For details regarding MIC determination, see Supporting Information, Figure S2).

Labeling was performed with glycerol-1,3-13C2. The cultivation medium was enriched with 2.41 µL glycerol-1,3-13C2 per mL medium (Sigma Aldrich, Germany) or as non-labeling control with 2.41 µL glycerol (Carl Roth, Germany) per mL. Concentration was determined as outlined in Figures S2 and S3. The cultivation was done at least in triplicates at 37°C, 140 rpm in glass tubes with an angle of 45°. At distinct time points, samples (500 µL) were taken and frozen at -20°C until extraction.

M. tuberculosis H37rv was cultivated with minor modifications. Instead of an overnight culture, a 7-day-old culture was used, diluted in fresh cultivation medium (Middlebrook 7H9, OADC, 0.05% Tween-80) with a starting OD600 of 0.2. The cultivation was done at least in triplicates at 37°C, 5 rpm in 5 mL Eppendorf caps at a rotary shaker.

Preparation of mycobacterial extract for MALDI-TOF analysis

The extraction of mycobacterial proteins was preceded as previously published.[15] The bacterial culture (300 µL) was filled into the well of filter plates (0.22 µm pore size, hydrophilic polypropylene, GHP). Bacterial cells were then immobilized onto the filter membrane by applying a vacuum until the growth medium was completely sucked through the membrane. After three consecutive washing steps with 300 µL of distilled H_2O with an incubation time of 5 min each, the filter plate was placed on ice, and 100 µL of ice-cold acetone was added and incubated for 5 min. Vacuum was used to remove the acetone through the chemically stable filter. To evaporate residual acetone sticking to the bottom of the plate, the filter plate was placed in a 37 °C incubator for 7 min on filter paper. Elution of the extracted protein was conducted by adding 20 µL of elution solution (50% acetonitrile, 35% formic acid, 15% distilled H_2O, v/v) to each well. After 5 min incubation at room temperature, a 96-well collection plate was placed below the filter plate and centrifuged at 4000 rpm for 3 min at 20 °C to collect the extracts.

Preparation of mycobacterial protein extract for LC-MS analysis

For each time point, different G*-labelled and non-labeled *M. smegmatis* culture volumes were chosen to guarantee an efficient amount of proteins within the sample. Culture volumes were 72 mL for 1.5 h, 45 mL for 4.5 h, 45 mL for 7.5 h, 36 mL for 12 h, and 27 mL for 24 h and were extracted as mentioned before. Protein extracts were combined, placed on ice, and evaporated under a nitrogen stream until an OD of 2.0 was reached. Those samples were directly injected into the LC-MS system, and fractions were collected every 30 s by placing a flow splitter (dividing the flow rate by half) directly in front of the sample inlet of the ESI. After MALDI-TOF verification of each collected fraction, the fractions (64, 77, 89, 93, 112, 117, 118, and 126) from two to four injections, including proteins of interest (3160.89 m/z, 4156.75 m/z, 4554.42 m/z, 5554.09 m/z, 9541.26 m/z, 4332.98 m/z, 5771.84 m/z, see Figure S6) were dried (Speed Vac), digested, and identified by Nano-LC-MS/MS.

Enzymatic digestion for protein identification via LC-MS/MS

To identify proteins of *M. smegmatis*, dried fractions were dissolved in 50 mM ammonium bicarbonate buffer containing 5 mM DTT and incubated for 30 min at 50˚C. Next, free cysteines were chemically modified by iodoacetamide (15 mM) for 30 min and subjected to proteolytic cleavage with 0.2 µg trypsin or LysC (Promega) for 14h at 30 ˚C. The resulting peptide fractions were desalted using C-18 stage tips, dried, and dissolved in 10 µL 0.1% formic acid for LC-MS/MS analysis.

2 SI Determination of minimal inhibitory concentration (MIC90)

For MIC90 determination, *M. smegmatis* was cultivated with different drug concentrations, and after overnight incubation (37 °C, 140 rpm), the OD_{600} was measured. The 1× MIC90 (90% of inhibition) values for *M. smegmatis* were determined to be 0.72 µg/mL for rifampicin, 0.52 µg/mL for linezolid, 10.33 ng/mL for bedaquiline and 4.73 ng/mL for BTZ043. For the determination of the reduction factors, the same samples were plated in serial dilution on Columbia agar (5% sheep blood), incubated at 37 °C for 3 days, and the colony-forming units (CFU)

were counted (Figure S2). The log reduction of viable mycobacteria after treatment was calculated as follows: reduction factor= $\log_{10}$ (number of viable mycobacteria before treatment) - $\log_{10}$ (number of viable mycobacteria after treatment).

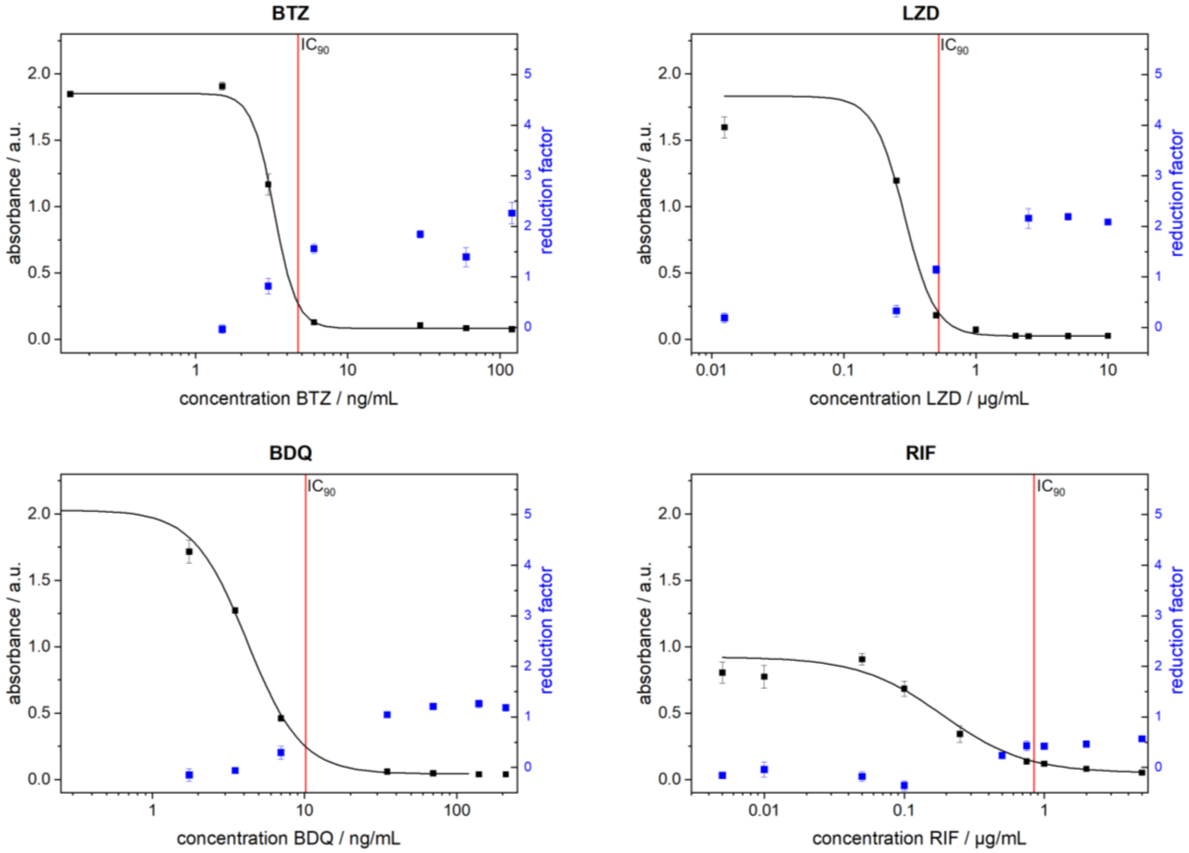

Figure S2: MIC- and reduction factor determination for BTZ, LZD, BDQ, and RIF.

3 SI   Choice of glycerol concentration

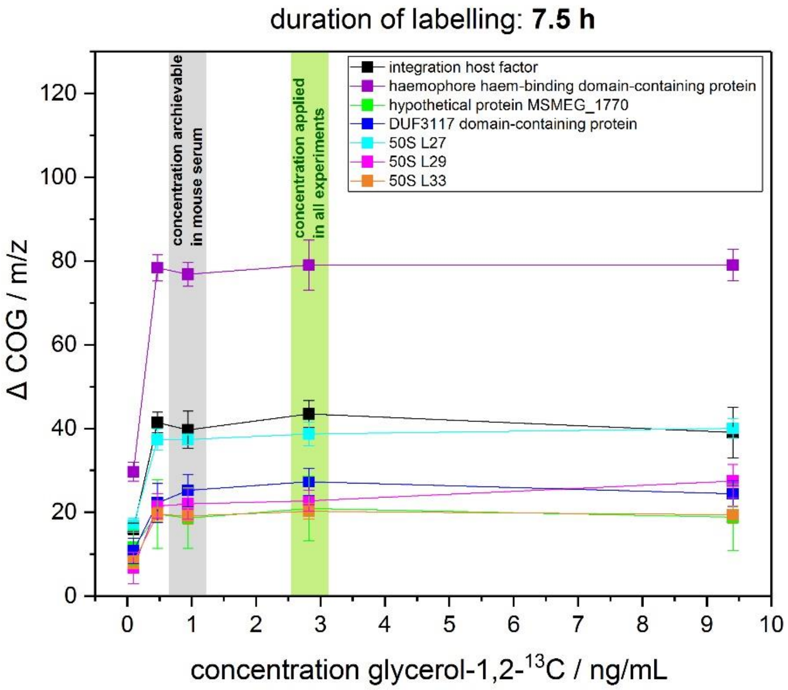

Figure S3: Saturation of differential center of gravity (ΔCOG) shift of seven identified protein mass peaks in *M. smegmatis* cultured in the presence of different G* concentrations. Between 0.47 and 9.40 ng/ml G* concentration, the COG saturated at constant levels for each peak. The lowest levels achievable in mouse serum are indicated with the grey bar.

4 SI   Labelling with 1,3-^{13}C labelled Glycerol (G*)

The *M. smegmatis* cultures were maintained in M7H9 supplemented with OADC. The cultures were grown under the presence of glycerol (G) or 1,3-^{13}C labeled glycerol (G*), respectively. A constant final concentration of 2.41 µl/mL glycerol was supplemented to the medium in different fractions between G and G* in the following increments: 33% G*, 50% G*, 66% G*, and 100% G*. Thereby, a linear dose-dependency of the main *de novo* synthesized peak can be observed. This is in line with the hypothesis that *de novo* synthesized protein will be almost instantaneously labeled to the extent expected with the amount of G* within the glycerol.

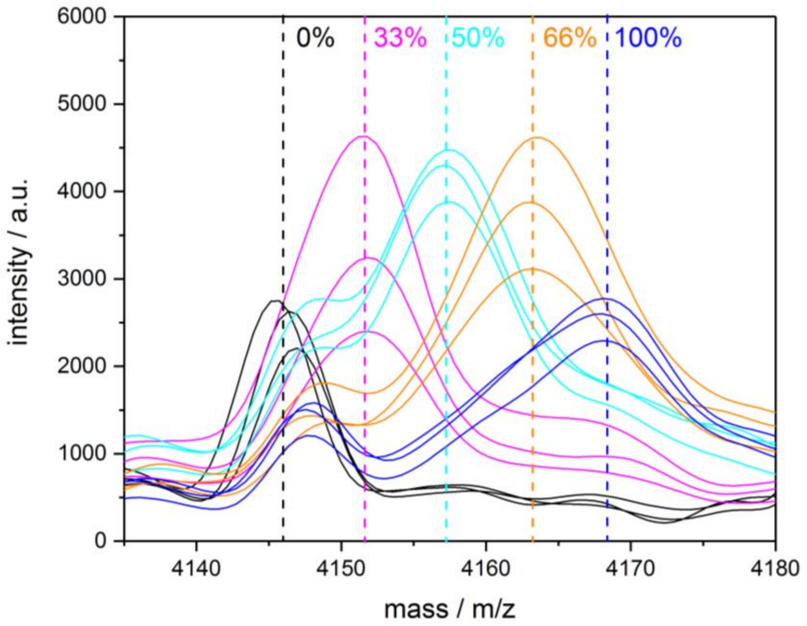

Figure S4: Discrete mass shifts with different G*/G quotients (same time-points, 1.5 h duration of labeling); Only G is represented as 0%, only G* is represented as 100%; Figure shows hypothetical protein MSMEG_1770 as an example.

To assess the linear concentration range in which labeling will be comparable, 1,3-$^{13}C_2$- labeled glycerol (G*) was added in increasing concentrations to mycobacterial cultures. ΔCOG shifts were measured for the seven chosen proteins for a concentration range between 0.094 and 9.40 ng/mL. Maximum ΔCOG shifts remain constant between 0.47 and 9.40ng/mL (Supplemental Figure S3). The concentration used within the study, 3.10 ng/mL (corresponds to 2.41 µL/mL) was chosen as it supports the growth of both strains used within the study (H37rv, mc^2155 equally well). To investigate whether the labeling of the proteins reaches saturation because of overall growth limitation, bacteria were grown in the presence of G*, and m/z shifts were investigated over time in parallel with the optical density (OD at 600 nm) (Figure S7). All proteins reach saturation long before the growth ceases due to high density, ruling out overall growth reduction as a reason for reduced further labeling. Besides, the chosen concentrations were sufficient to supply so much ^{13}C that even after prolonged growth for 24 h in the case of *M. smegmatis* and 168 h in the case of *M. tuberculosis*, the remaining concentration is still sufficient to maintain full labeling for growing bacteria within the determined concentrations. The presence of sufficient amounts of G* was experimentally further verified by harvesting spent G* containing media after 24h of growth in the case of *M. smegmatis* and sterile filtering it. The filtered media was then again used to start a culture for an additional 24 h. While final OD_{600} values remained unchanged, the extent of maximum observed ΔCOG shifts for the proteins was also equal to the shifts observed in medium with freshly added G* in the concentration of 2.7ng/ml.

5 SI MALDI-TOF verification via HPLC-ESI-Orbitrap

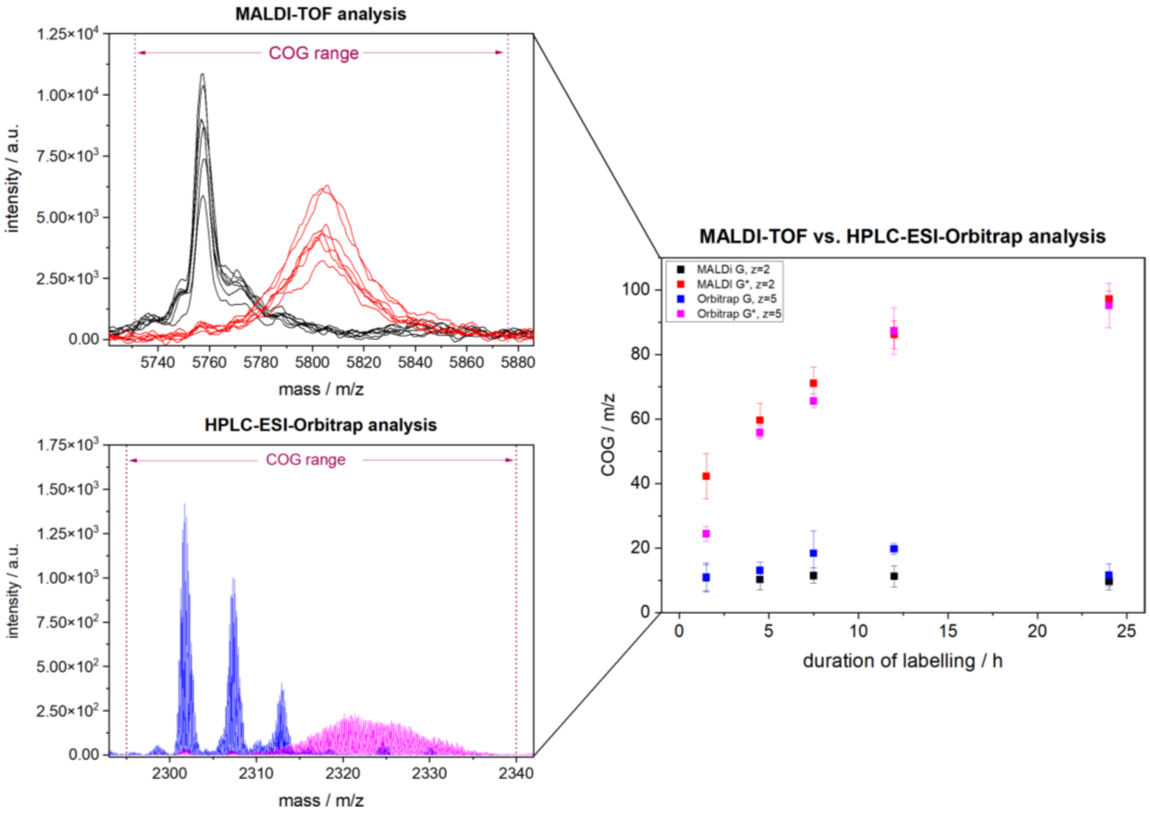

Figure S5: Left side: MALDI-TOF MS vs. HPLC-ESI-Orbitrap MS measurement of labelled (red, magenta) and unlabelled integration host factor (black, blue). Right side: COG analysis of a chronological labelling sequence (5 time points) of integration host factor.

6 SI Fraction collection for protein identification

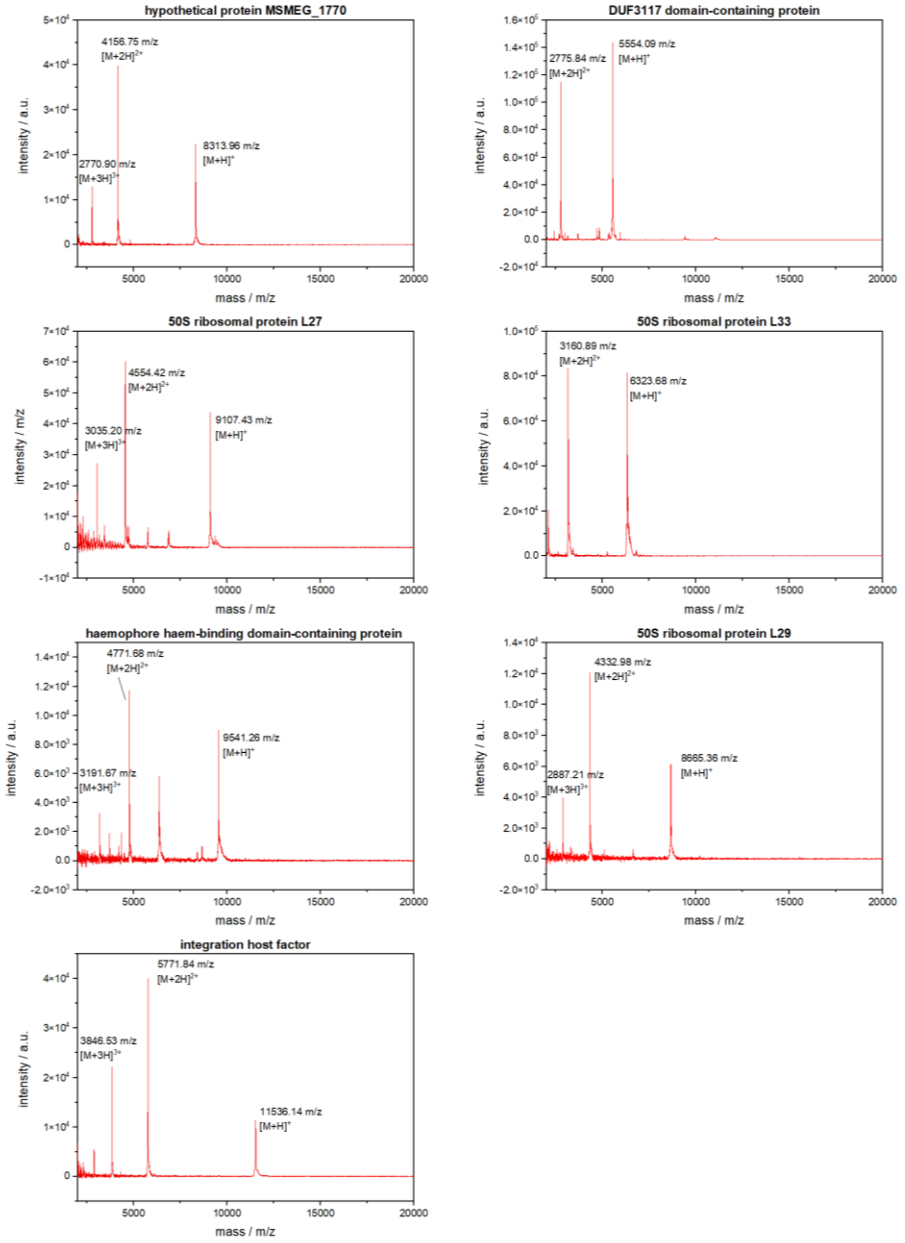

Figure S6: MALDI-TOF spectra for isolated proteins (3160.89 m/z, 4156.75 m/z, 4554.42 m/z, 5554.09 m/z, 9541.26 m/z, 4332.98 m/z, 5771.84 m/z) derived from fraction collection ever 30 s after injection of a *M. smegmatis* protein extract.

7 SI Turnover of mature mycobacteria proteins

Four potential explanations may be compatible with the fast COG saturation: First, the growth of the culture might cease, and thus, no further incorporation takes place. This can be excluded by optical density measurements, which show a constant rise in optical density with a still-rising OD_{600} of 4.7 being reached after 25 h. Second, the concentration of available G* might become limiting over time. However, we do not observe changes in saturation levels by varying the G* concentrations between 0.5 and 9.4 ng/ml (see Supplemental Figure 3) or by re-culturing *M. smegmatis* in a filtered medium with an initial G* concentration of 2.7 ng/ml, which had already been used to culture a previous *M. smegmatis* culture for 16 h (data not shown), thus excluding this explanation. The third

possible explanation is the use of other carbon sources over time. This, however, can be excluded as we observe different saturation times for the different proteins. For example, the protein at m/z 4150 saturates already after ~2.5 h (light green in Figure 4), while all other proteins investigated in this study saturated after 10.5 h and later. Fourth, if changed metabolic pathways would reduce the incorporation of ^{13}C from G*, the analytes/metabolites *newly* synthesized after the said change would have reduced m/z shifts. This effect should be detectable in the case of changed metabolism as growth continues for a prolonged time (see OD_{600} measurements (dashed red line) in figure 4), but was not observed either.

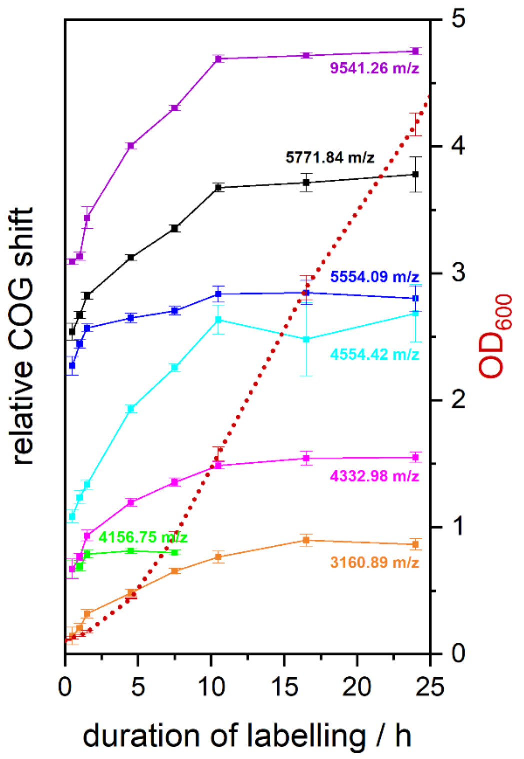

Figure S7: COG shift of peaks at different m/z values (orange: 3160.89 m/z, green: 4156.75 m/z, pink: 4332.98 m/z, cyan: 4554.42 m/z, blue: 5554.09 m/z, black: 5771.84 m7z, purple: 9541.26 m/z) coming from *M. smegmatis* bacteria cultured in the presence of G* over time. Parallel measurements of OD_{600} (dashed red line) representing bacterial growth.

The general principles of protein biosynthesis starting from the amino acid pool, however are similar for all proteins; hence, all proteins produced at each specific time-point are derived from the same relative composition of labeled and unlabeled precursor building blocks. Within the rich media used in the study, no free amino acids are present, thus the direct influx of unlabeled amino acids is unlikely. The incorporation of ^{13}C into the amino acid pool seems to happen almost instantaneously after the addition of G*(see Figure S7, e.g. green line corresponding to 4156.75 m/z, identified as hypothetical protein MSMEG_1770). Due to this, the kinetics of the labeling is highly stable and reproducible; the overall ^{13}C contribution, however, may vary between the products. This argues again that potential other, slower metabolic pathways potentially leading to additional labeling are insignificant under the chosen conditions.

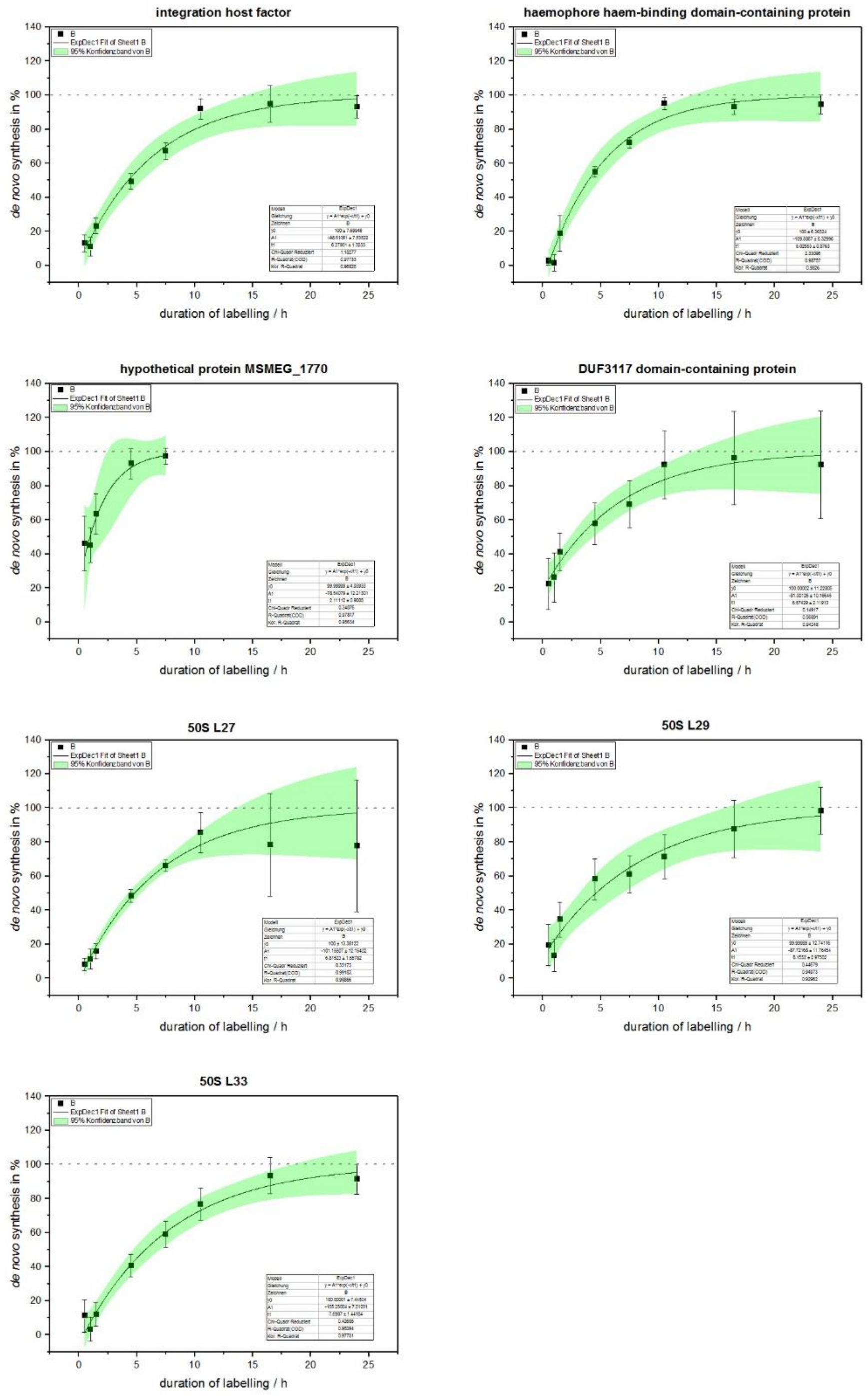

Figure S8: Labelling dynamics arrived from ΔCOG (using exponential decay fit with 95 % confidence band) for seven identified *M. smegmatis* proteins (integration host factor, haemophore haem-binding domain-containing protein, hypothetical protein MSMEG_1770, DUF3117 domain-containing protein, 50S L27, 50S L29, 50S L33) within 24 hours (timepoints for sampling: 0.5, 1.0, 1.5, 4.5, 7.5, 10.5, 16.5 and 24 h). Each the upper amplitude was set to 100 % of *de novo* protein synthesis.

8 SI *De novo* protein synthesis analysis reveals the early onset of antibiotic actions

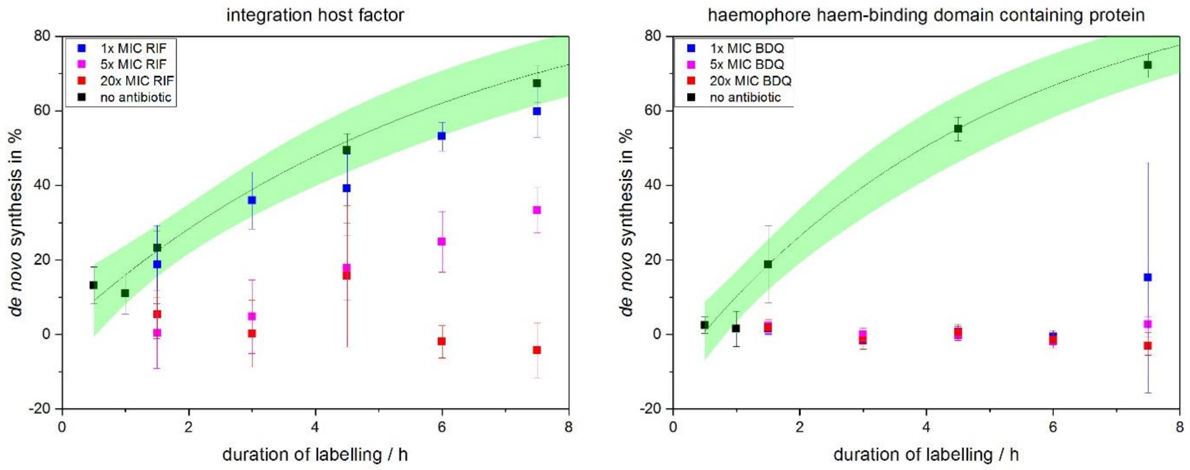

Figure S9: *De novo* protein synthesis in % exemplified for left: integration host factor under the stressor of 1x (blue), 5x (magenta), and 20x (red) MIC RIF and right: haemophore haem-binding domain containing protein under the stressor of 1x (blue), 5x (magenta) and 20x (red) MIC BDQ each within the first 7.5 h of antibiotic treatment.

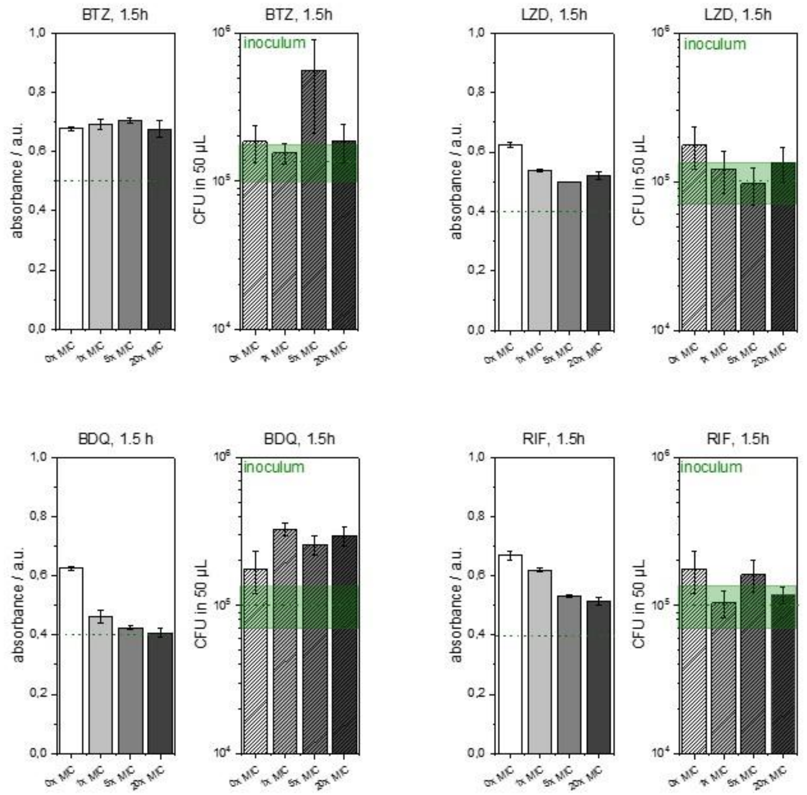

Figure S10: OD_{600} measurements and the corresponding CFU counts for *M. smegmatis* cultures incubated with BTZ, LZD, BDQ or RIF, each in 3 different MICs (1x, 5x, 20x) after 1.5 h of incubation; the green bar to the right of the CFU bar chart represents the inoculation CFU number for the respective condition (mean +/- SD).

9 SI Antibiotic effects over time/dynamics

The stop of protein synthesis of specific proteins, or the majority of proteins, is highly interesting to elucidate the dynamics of bacterial killing by antimicrobials, especially in microorganisms such as mycobacteria, which are known to be notoriously hard to kill. Due to the different production rates of individual proteins, a saturation of labeling for individual proteins will occur at different time points. The faster a protein is turned over and produced anew, the quicker the ΔCOG will reach saturation. Shifting of protein COG back towards lower masses is not possible as long as sufficient G* is available, as it would require a selective metabolism of unlabelled G. Under the chosen conditions in this study, sufficient G* is present at all times (see Supporting Information, chapter SI 4) after starting the labeling procedure. Saturated ΔCOGs do not allow the detection of changes in production rates anymore, thus limiting the meaningful observation time. To overcome this limitation, the addition of G* can be delayed to start the labeling process later and thus detect alterations in production rates at later time points while maintaining high sensitivity for changes. Staggering timelines like this allow for covering longer observation periods (see Figure 5). Using three staggered starts, at 0 h, 3 h, and 6 h for each condition and group, respectively, with individual labeling periods of 7.5 h allows covering a total observation period of 13.5 h in *M. smegmatis*.

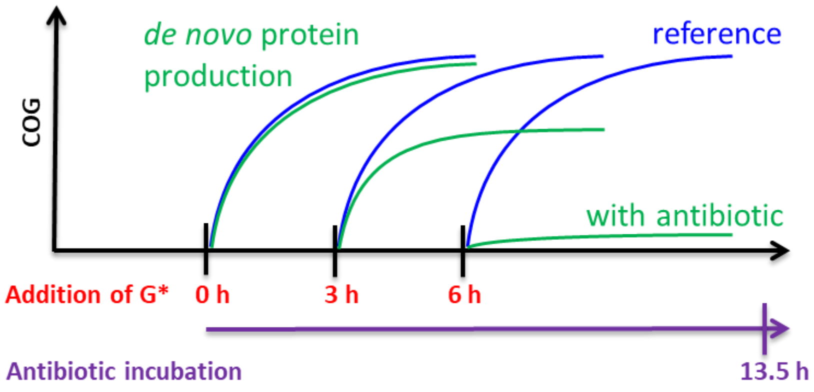

Figure S11: Schematic of staggered timelines principle. Single antibiotics (BTZ, LZD, BDQ, or RIF, each in 1x, 5x and 20x MIC) were added at the beginning (0 h) and G* at the beginning (0h), after 3 h and 6 h of antibiotic incubation.

10 SI Antibiotic effect on *M. tuberculosis*

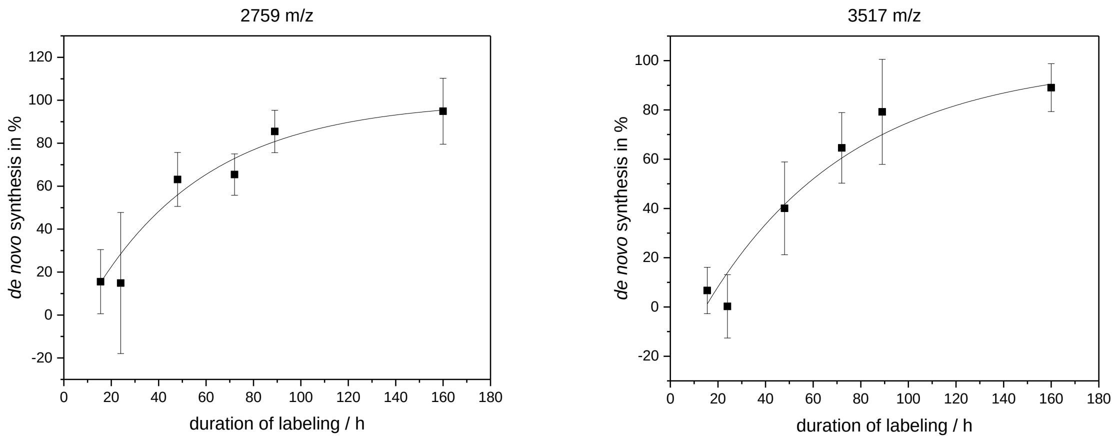

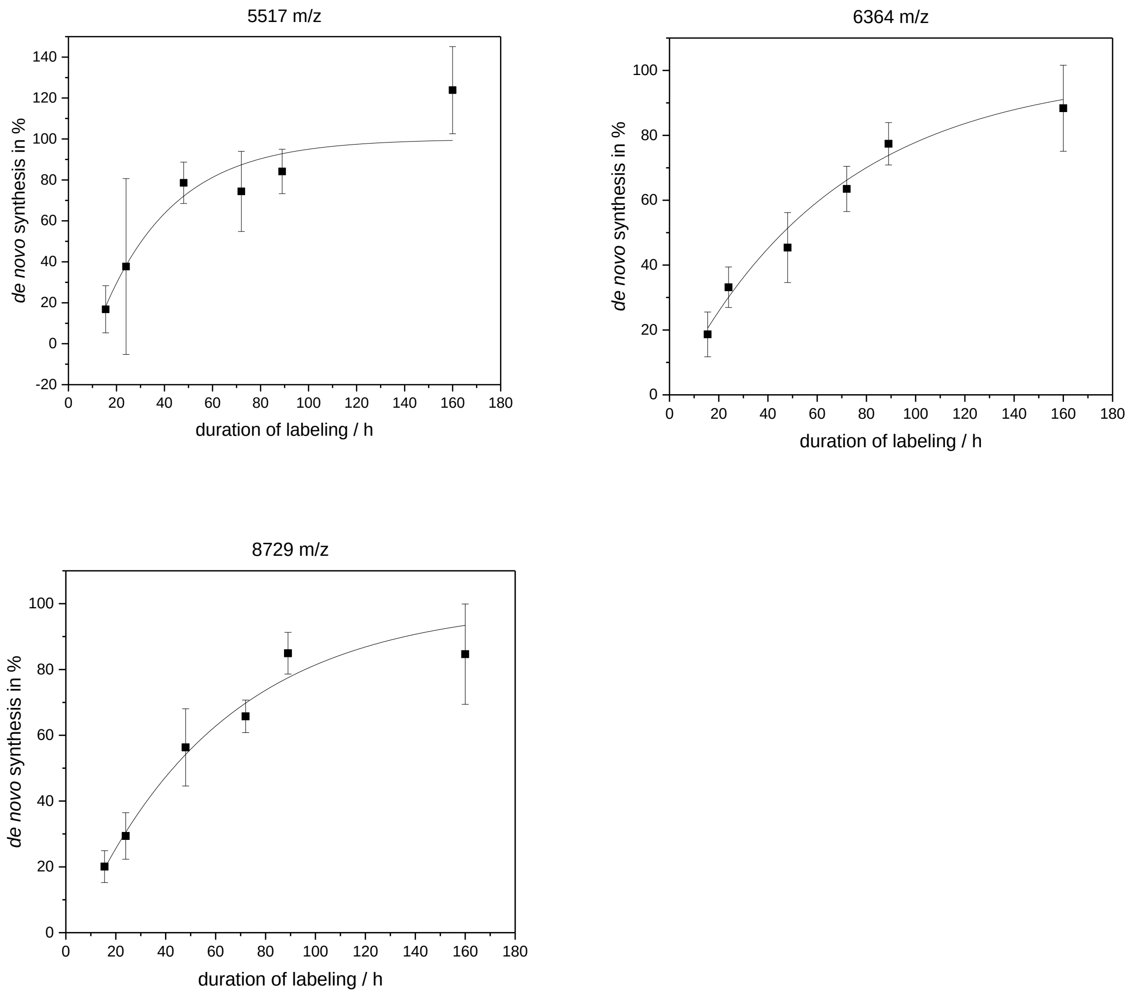

Figure S12: Modelled labeling kinetics of five chosen analytes for *M. tuberculosis* H37rv under normal growth conditions in rich media.

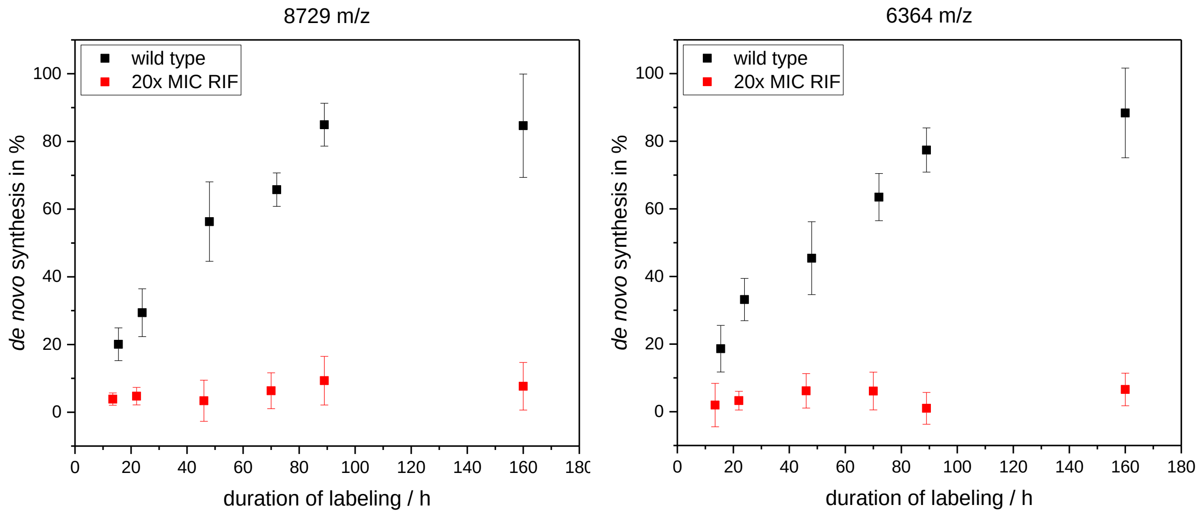

Figure S13: Change of labeling kinetics over time for *M. tuberculosis* H37rv analytes (6364 m/z and 8729 m/z) after addition of 20x MIC RIF and different times of incubation.
